# Dosing, treatment patterns and safety of finerenone use in routine care: an interim analysis of the prospective, real-world and observational FINE-REAL study

**DOI:** 10.1093/ckj/sfaf305

**Published:** 2025-10-06

**Authors:** David C Wheeler, Kevin M Pantalone, Lixin Guo, Nihar R Desai, Ricardo Correa-Rotter, Susanne B Nicholas, Nan Hee Kim, Zihe Zheng, Charlie Scott, Andrea Horvat-Broecker, Martin Merz, Christoph Wanner, Sankar D Navaneethan

**Affiliations:** Centre for Kidney and Bladder Health, University College London, London, UK; Department of Endocrinology, Cleveland Clinic, Cleveland, OH, USA; Department of Endocrinology, Beijing Hospital, National Center of Gerontology, Institute of Geriatric Medicine, Chinese Academy of Medical Sciences, Beijing, China; Section of Cardiovascular Medicine, Yale School of Medicine, Yale New Haven Hospital, New Haven, CT, USA; Department of Nephrology and Mineral Metabolism, Instituto Nacional de Ciencias Médicas y Nutrición Salvador Zubirán, Mexico City, Mexico; Department of Medicine, Division of Nephrology, David Geffen School of Medicine at University of California, Los Angeles, Los Angeles, CA, USA; Division of Endocrinology and Metabolism, Department of Internal Medicine, Korea University College of Medicine, Seoul, Republic of Korea; US Medical Affairs – Cardiovascular-Renal, Bayer US LLC, Whippany, NJ, USA; Clinical Statistics and Analytics, Bayer US LLC, Whippany, NJ, USA; Medical Affairs and Pharmacovigilance, Bayer AG, Wuppertal, Germany; Global Medical and Evidence Cardiology, Bayer AG, Berlin, Germany; Department of Clinical Studies and Epidemiology, Comprehensive Heart Failure Center, University Hospital Würzburg, Würzburg, Germany; Section of Nephrology, Baylor College of Medicine, Houston, TX, USA

**Keywords:** chronic kidney disease, FINE-REAL, finerenone, observational study, type 2 diabetes

## Abstract

**Background:**

Finerenone improves cardiovascular and renal outcomes in patients with type 2 diabetes (T2D) and chronic kidney disease (CKD), but real-world data are limited. The FINE-REAL study (NCT05348733) is examining the use of finerenone (10 or 20 mg) in participants with CKD and T2D in routine clinical practice.

**Methods:**

FINE-REAL is an ongoing global, prospective, single-arm, non-interventional study. This preplanned interim analysis was conducted 2 years after the first participant first visit. Assessments included demographics, concomitant medications and safety.

**Results:**

Data were available for 1916 participants with a median age of 68 years [interquartile range (IQR) 59–74], 65% male, with a median follow-up of 260 days (IQR 139–357). The baseline mean estimated glomerular filtration rate (Chronic Kidney Disease Epidemiology Collaboration equation) was 54 ml/min/1.73 m^2^ (standard deviation 24) and the median urine albumin:creatinine ratio was 293 mg/g (IQR 86–783). Prior/concomitant medications included renin–angiotensin system inhibitors (73%), sodium–glucose cotransporter 2 inhibitors (53%) and glucagon-like peptide 1 receptor agonists (29%). Finerenone 10 mg/day was initiated in 1548 (81%) participants and 367 (19%) were initiated on 20 mg/day. During follow-up, 404 of the 1548 participants starting at 10 mg (26%) increased their dose to 20 mg. Finerenone was continuously administered for up to 1 year in 85% of participants, interrupted in 6% and discontinued in 13%. The most common adverse events were hyperkalaemia (8% of participants; leading to discontinuation in 1% and hospitalization in 0.3%; fatal in none), urinary tract infection (4%) and urogenital tract haemorrhage (including haematuria) (3%).

**Conclusions:**

In this real-world population, most participants initiated finerenone at 10 mg and remained on that dose. Finerenone was well tolerated and safety was consistent with the known profile of the drug. This interim analysis and future data from FINE-REAL will help to guide decision-making regarding the use of finerenone in participants with CKD and T2D.

## Graphical Abstract

**Graphical Abstract:**
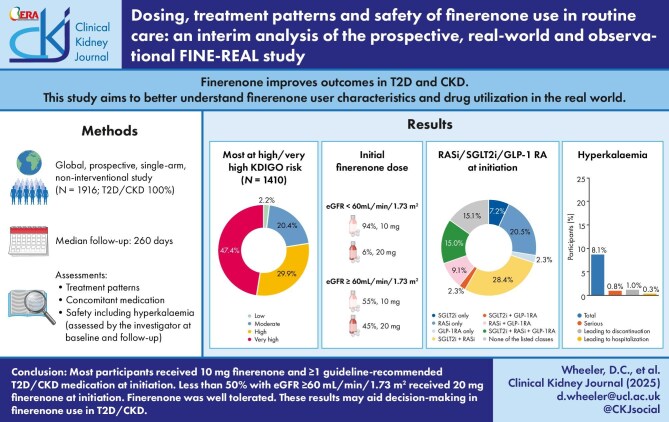

KEY LEARNING POINTS **What was known:**

- Finerenone has been shown to improve cardiovascular and renal outcomes in patients with chronic kidney disease (CKD) and type 2 diabetes (T2D).
- Real-world data on the use of finerenone remain limited.

**This study adds:**

- FINE-REAL is the first global, prospective, observational study investigating the use of finerenone in participants with CKD and T2D in routine clinical care; this interim analysis included 1916 participants with a median follow-up of 9 months.
- Most participants initiated finerenone at 10 mg and remained on that dose; titration to 20 mg, as recommended on the label for most participants, was uncommon.
- Finerenone was well tolerated and safety was consistent with the known profile of the drug.

**Potential impact:**

- This interim analysis will help to improve informed decision-making regarding initiation and dosing of finerenone in patients with CKD and T2D.

## INTRODUCTION

Chronic kidney disease (CKD) and type 2 diabetes (T2D) contribute significantly to morbidity and mortality worldwide [1–4]. Recent estimates suggest that CKD affects ≈10% of the global population [5] and ≈13% of adults have T2D [6]. Co-occurrence of CKD and T2D is common—≈40% of patients with T2D develop CKD [7]—and it predisposes to cardiovascular (CV) events [1, 8–10]. Patients with T2D and CKD have ≈40% higher risks of CV disease and all-cause mortality than those with T2D alone [11, 12]. CV disease is the leading cause of death in patients with CKD regardless of CKD progression [13]. Causes of CV diseases in patients with CKD and T2D include metabolic abnormalities, hypertension, prior CV events, inflammation, endothelial dysfunction and oxidative stress [12, 14–18]. In addition, albuminuria and decreased estimated glomerular filtration rate (eGFR) are independent predictors of CV mortality [19, 20].

Patients with CKD and T2D have overactivation of mineralocorticoid receptors, which induces inflammation, fibrosis and oxidative stress in endothelial cells and vascular smooth muscle cells, increasing the risk of kidney and CV diseases [21–23]. In preclinical and phase 2 studies, the non-steroidal, selective mineralocorticoid receptor antagonist (MRA) finerenone improved markers of kidney and CV damage [8, 24–28]. Finerenone was subsequently assessed in two randomized phase 3 trials in patients with T2D and CKD: FIDELIO-DKD (NCT02540993) [8] and FIGARO-DKD (NCT02545049) [29]. In FIDELIO-DKD, progression of kidney disease (kidney failure, sustained ≥40% decrease in eGFR from baseline over ≥4 weeks or renal death) was reduced by 18% with finerenone versus placebo. In FIGARO-DKD, the primary composite CV outcome (death from CV causes, non-fatal myocardial infarction, non-fatal stroke or hospitalization for heart failure) was reduced by 13% with finerenone versus placebo [25, 26, 29]. The FIDELITY study, a prespecified pooled analysis of FIDELIO-DKD and FIGARO-DKD, concluded that, compared with placebo, finerenone significantly reduced composite kidney and CV outcomes [26]. Finerenone is approved for the treatment of CKD associated with T2D [30, 31] and is recommended in national and international guidelines [32, 33]. Despite clinical trial results and guideline recommendations, real-world data on the use of finerenone remain limited.

FINE-REAL (NCT05348733) is a global, prospective, single-arm, non-interventional study evaluating the use of finerenone in participants with CKD and T2D in routine clinical practice [34]. Initiated in June 2022, FINE-REAL is expected to be completed in August 2026, except for South Korea, where the recruitment is still ongoing. The first interim analysis of 556 participants has been published [35]. Here we present a prespecified interim analysis at 2 years after the first participant first visit (cut-off date: 13 June 2024), describing participant characteristics, dosing regimens, treatment patterns and the safety of finerenone.

## MATERIALS AND METHODS

### Objectives

The primary objective of this interim analysis was to describe treatment patterns, i.e. dosing regimens and concomitant medication in participants with CKD and T2D treated with finerenone. Assessments included the dose and duration of finerenone treatment and the use of concomitant therapies. Secondary assessments included the occurrence, frequency and severity of treatment-emergent adverse events (TEAEs) and serious TEAEs, with a focus on hyperkalaemia. Hyperkalaemia outcomes included those leading to permanent discontinuation of finerenone, dialysis, hospitalization or death.

### Study participants

Eligible participants were ≥18 years of age with a diagnosis of CKD associated with T2D, based on physician assessment, and receiving finerenone (10 or 20 mg orally once daily) as part of routine clinical practice in accordance with local marketing authorization. Participants were only enrolled after the decision to initiate finerenone treatment or if they were already on finerenone. Participants could only be included in the study within a time window of 8 weeks before or after the initiation of finerenone. All study participants provided written informed consent before entering this ongoing study, which is being conducted in accordance with the principles of the Declaration of Helsinki. Protocol approvals were obtained from local regulatory authorities and the ethics committees of participating centres.

### Study follow-up

All follow-up visits were documented, but given the observational nature of the study, there was no fixed schedule for these visits. The observation period for each participant ended 12 months after the start of finerenone treatment. If cessation of finerenone therapy or death occurred before 12 months, patients were censored at that time, plus an additional 30 days to record TEAEs.

### Assessments

Demographic characteristics and disease history were recorded at baseline. Baseline measurements included all entries in the electronic clinical research form with no restriction on time of assessment. In some participants, no data were available from before finerenone initiation, and baseline measurements were taken after the start of finerenone. Concomitant medications, prespecified concomitant diseases, laboratory parameters [eGFR and urine albumin:creatinine ratio (UACR)], adverse events (AEs) and hyperkalaemia assessments were recorded at baseline and follow-up visits [34]. Prespecified concomitant diseases included myocardial infarction, acute coronary syndrome, stroke, transient ischaemic attack, limb ischaemia, claudication, ulcers of the extremities, sepsis, heart failure, atrial fibrillation, supraventricular tachycardia, hypertension, diabetic retinopathy and hyperkalaemia. Concomitant medications of interest were defined as treatments for CKD associated with T2D, i.e. renin–angiotensin system inhibitors (RASis), sodium–glucose cotransporter 2 inhibitors (SGLT2is) and glucagon-like peptide 1 receptor agonists (GLP-1RAs). eGFR was recorded according to the investigator’s calculation methods, but this study used the Chronic Kidney Disease Epidemiology Collaboration (CKD-EPI) equation without race formula [36, 37] for calculations of eGFR values [38]. Kidney Disease: Improving Global Outcomes (KDIGO) risk categories [39] were calculated for all participants for whom data were available.

TEAEs are AEs that happened during finerenone therapy regardless of any potential relationship to treatment and were reported according to Medical Dictionary for Regulatory Activities (MedDRA) definitions [40]. Hyperkalaemia-related events were reported as TEAEs with no defined potassium threshold except at the discretion of the investigator. Similar to FIDELIO-DKD [8, 41] and FIGARO-DKD [29], the definition of hyperkalaemia included the MedDRA preferred terms ‘hyperkalaemia’ and ‘blood potassium increased’. The definition of hyperkalaemia in FINE-REAL was similar to that in FIDELIO-DKD [8, 41] and FIGARO-DKD [29].

### Statistical analysis

Electronic records were used for data capture and were validated according to US Food and Drug Administration regulations [42]. Statistical analyses were exploratory and descriptive in nature.

All data are presented for the full analysis set (FAS), defined as all participants who signed the informed consent form and received at least one dose of finerenone during the observation period. Participant demographics and other characteristics, prior and concomitant medication, safety and outcomes of interest (including hyperkalaemia-related events and diabetic retinopathy) were described by frequency tables and summary statistics. The cumulative incidence of hyperkalaemia TEAEs was calculated using Aalen–Johansen estimates [43], with 95% confidence intervals.

## RESULTS

### Participant characteristics

**Figure 1:**
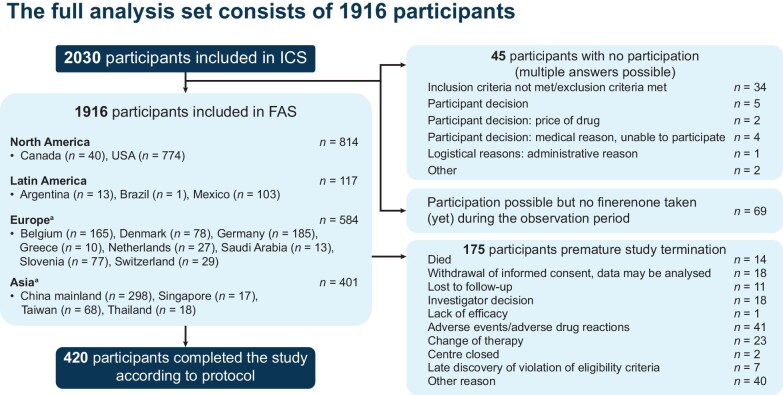
Study flow diagram. ^a^Recruitment has begun in Portugal and South Korea, but data are not available for the current analysis. ICS: informed consent analysis set.

Participant disposition is shown in Fig. 1. At the cut-off date, the median follow-up time was 259.5 days [interquartile range (IQR) 138.5–357.0]. Of 2030 participants enrolled across 19 countries, 45 were unable to participate due to inclusion and/or exclusion criteria violations, participant decision, physician decision or other reasons and another 69 were excluded because they had not yet received finerenone at the time of data cut-off. Therefore, 1916 participants were included in the FAS, including 774, 298, 185, 165 and 103 from the USA, China, Germany, Belgium and Mexico, respectively.

The baseline median age was 68 years (IQR 59–74) and the mean age was 66.5 years [standard deviation (SD) 11.1]. The median duration of T2D was 14 years (IQR 8–22). Overall, 1769 (92.3%) participants were ≥50 years of age and 1243 (64.9%) participants were male (Table 1).

**Table 1:** Baseline demographics and disease characteristics (FAS, *n* = 1916).

Characteristic	Values
Age (years), mean (SD)	66.5 (11.1)
Sex, *n* (%)	
Male	1243 (64.9)
Female	673 (35.1)
BMI (kg/m^2^), median (IQR) (*n* = 1614)	29.8 (25.8–34.5)
Race or ethnic group, *n* (%)	
White	993 (51.8)
Asian	500 (26.1)
Black/African American	165 (8.6)
Other^a^	22 (1.1)
Not reported	236 (12.3)
Time from diagnosis of T2D (years), median (IQR) (*n* = 1631)	14.0 (8.0–22.0)
Time from diagnosis of CKD (years), median (IQR) (*n* = 1550)	4.0 (2.0–7.0)
Serum potassium (mmol/l), median (IQR) (*n* = 1783)	4.4 (4.1–4.7)
Serum potassium (mmol/l), *n* (%) (*n* = 1783)	
≤3.6	86 (4.8)
>3.6–5.0	1611 (90.4)
>5.0–5.5	71 (4.0)
>5.5–6.0	12 (0.7)
>6.0	3 (0.2)
HbA1c (%), mean (SD) (*n* = 1027)	7.5 (1.5)
Seated systolic blood pressure (mmHg), mean (SD) (*n* = 1737)	136.7 (19.0)
Seated diastolic blood pressure (mmHg), mean (SD) (*n* = 1736)	77.0 (10.9)

a American Indian or Alaska Native, Native Hawaiian or other Pacific Islander.

BMI: body mass index; HbA1c, glycated haemoglobin.

**Figure 2:**
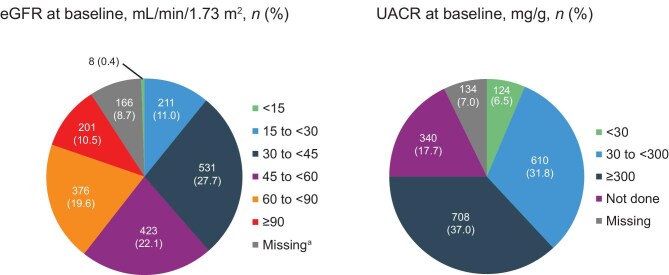
eGFR and UACR measurements at baseline. Not done: assessment not performed according to physician’s practice. eGFR was derived from the CKD-EPI equation without race (calculated). ^a^Missing: data not entered or collected.

Baseline eGFR and UACR data were available for 1750 (91.3%) and 1442 (75.3%) participants, respectively (Supplementary Table S1). The mean eGFR [CKD-EPI without race (calculated)] was 54.4 ml/min/1.73 m^2^ (SD 24.2). The median UACR at baseline was 292.7 mg/g (IQR 85.8–783.4) and 124 (6.5%) participants had a UACR <30 mg/g (Fig. 2). At baseline, 1611/1783 (90.4%) participants with data available had a serum potassium of 3.6–5.0 mmol/l.

Prior and concomitant diseases (prespecified) were reported in 1875 (97.9%) participants, the most common being hypertension [1678 (87.6%)], atrial fibrillation [131 (6.8%)] and chronic heart failure [125 (6.5%)]. Hyperlipidaemia and dyslipidaemia, which were not prespecified, were reported in 729 (30.0%) and 294 (15.3%) participants, respectively. Complications related to T2D were present in 808 (42.2%) participants, with diabetic retinopathy [300 (15.7%)], diabetic neuropathy [284 (14.8%)] and coronary artery disease [204 (10.6%)] being the most common.

Proliferative diabetic retinopathy was present in 52 (2.7%) participants at baseline. Among 116 (6.1%) participants with non-proliferative diabetic retinopathy (NPDR), 41 (2.1%), 42 (2.2%) and 19 (1.0%) were diagnosed with mild, moderate or severe disease, respectively. Information on NPDR grade was unknown or missing in 17 (0.9%) participants.

**Figure 3:**
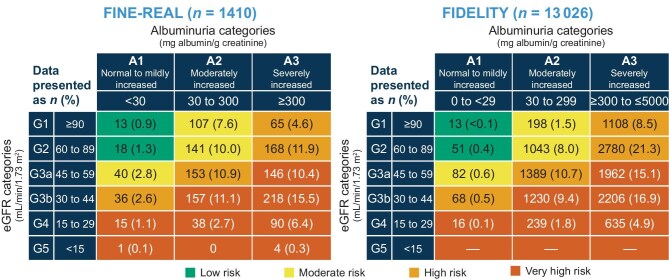
KDIGO risk categories at finerenone baseline in FINE-REAL and FIDELITY.

At baseline, data for both eGFR and UACR for calculation of KDIGO risk were available for 1410 (73.6%) participants. In these 1410 participants, the proportions in the low-, moderate-, high- and very high-risk KDIGO groups were 2.2% (*n* = 31), 20.4% (*n* = 288), 29.9% (*n* = 422) and 47.4% (*n* = 669), respectively, compared with 0.5% (*n* = 64), 10.2% (*n* = 1323), 41.0% (*n* = 5245) and 48.3% (*n* = 6288), respectively, in FIDELITY (Fig. 3).

### Dosing and treatment patterns

**Figure 4:**
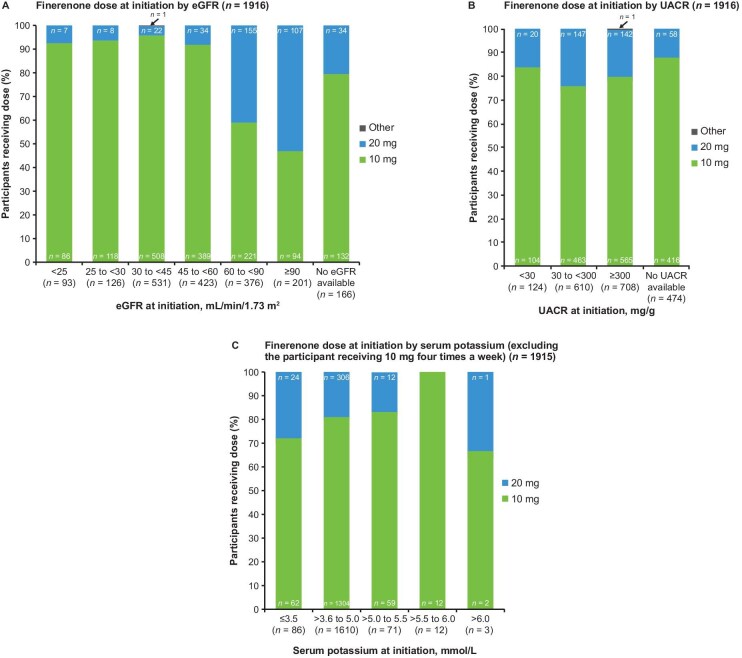
Finerenone dose at baseline by **(A)** eGFR, **(B)** UACR and **(C)** serum potassium.

Overall, 1548 (80.8%) participants initiated treatment with daily finerenone 10 mg, 367 (19.2%) with 20 mg and 1 (0.1%) with another dose/frequency (10 mg finerenone four times per week). Fig. 4 shows the relationship between initial finerenone dose and eGFR (Fig. 4A), UACR (Fig. 4B) and serum potassium (Fig. 4C).

**Figure 5:**
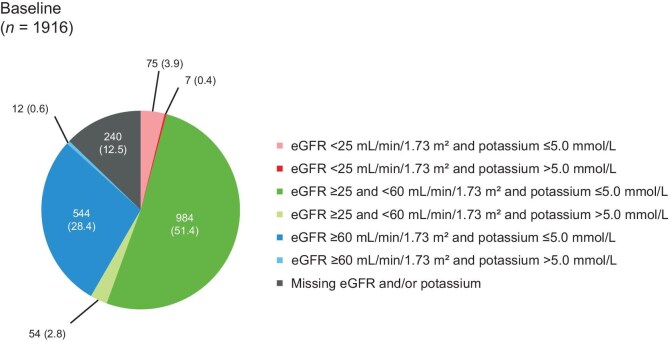
eGFR and potassium levels at baseline. Data presented as *n* (%). Baseline is defined as the availability of eGFR and potassium level at any time before the start day of finerenone.

Overall, 577/1916 (30.1%) participants had a baseline eGFR >60 ml/min/1.73 m^2^, and of these, 262 (45.4%) were initiated with 20 mg finerenone. The distribution of eGFR and serum potassium is shown in Fig. 5 and the starting doses in these groups are shown in Table 2. Across categories of UACR, 104 (5.4%)–565 (29.5%) participants received finerenone 10 mg at baseline (Fig. 4). As baseline serum potassium category levels increased (≤3.6, 3.6–5.0 and >5.0–5.5 mmol/l), the proportion of participants receiving 20 mg of finerenone decreased. The sample sizes for baseline serum potassium levels >5.5 mmol/l were too small for meaningful analysis.

**Table 2:** eGFR and potassium at baseline stratified by starting dose of finerenone (FAS).

Characteristics	*n*	10 mg, *n* (%)	20 mg, *n* (%)
Overall^a^	1916	1548 (80.8)	367 (19.2)
eGFR <25 ml/min/1.73 m^2^ and potassium ≤5.0 mmol/l	75	70 (93.3)	5 (6.7)
eGFR <25 ml/min/1.73 m^2^ and potassium >5.0 mmol/l	7	7 (100.0)	0 (0.0)
eGFR ≥25–<60 ml/min/1.73 m^2^ and potassium ≤5.0 mmol/l^a^	983	928 (94.4)	55 (5.6)
eGFR ≥25–<60 ml/min/1.73 m^2^ and potassium >5.0 mmol/l	54	48 (88.9)	6 (11.1)
eGFR ≥60 ml/min/1.73 m^2^ and potassium ≤5.0 mmol/l	544	298 (54.8)	246 (45.2)
eGFR ≥60 ml/min/1.73 m^2^ and potassium >5.0 mmol/l	12	6 (50.0)	6 (50.0)
Missing eGFR and/or potassium	240	191 (79.6)	49 (20.4)

a One participant in the eGFR ≥25–<60 ml/min/1.73 m^2^ and potassium ≤5.0 mmol/l group (0.1% of this group and 0.05% of the total population) received another dose.

Over 12 months of follow-up, the proportion of participants receiving finerenone 10 mg decreased and the proportion receiving 20 mg increased (Supplementary Fig. S1). An eGFR value after initiation of finerenone was available for 1477 (77.1%) participants and was accompanied by a potassium value for at least one visit in 1464 (76.4%). Overall, 404/1548 (26.1%) participants who started with finerenone 10 mg underwent up-titration of their dose to 20 mg after a median of 47.0 days (IQR 30.0–108.5), while 28/367 (7.6%) who started at 20 mg underwent down-titration after a median of 58.0 days (IQR 30.0–139.0). The reasons for down-titration are shown in Table 3. In total, 771/1916 (40.2%) participants received finerenone 20 mg at some time during the study. Potassium >5.5 mmol/l was detected in 32 (4.2%) of these participants, of whom 20 (62.5%) underwent down-titration. Of 1548 participants initiating finerenone at 10 mg, 1196 (77.3%) had at least one assessment of eGFR and potassium at initiation and follow-up. At any time during follow-up, 683/1548 (44.1%) participants had an eGFR reduction ≤30% and potassium ≤4.8 mmol/l (the level at which increasing the dose to 20 mg is recommended); 289/1548 (18.7%) had an eGFR reduction ≤30% and potassium >4.8 mmol/l; 50/1548 (3.2%) had an eGFR reduction >30% and potassium ≤4.8 mmol/l; and 34/1548 (2.2%) had an eGFR reduction >30% and potassium >4.8 mmol/l. The rates of up-titration during follow-up in these four groups were 210/683 (30.7%), 31/289 (10.7%), 4/50 (8.0%) and 0/34 (0%), respectively.

**Table 3:** Reasons for down-titration from finerenone 20 mg to 10 mg.

Reason	Participants, *n* (%)
Overall	28 (100.0)
Adverse event	8 (28.6)
Change in eGFR	5 (17.9)
Change in serum potassium	5 (17.9)
Physician decision	5 (17.9)
Other reason	3 (10.7)
Participant decision	2 (7.1)

**Figure 6:**
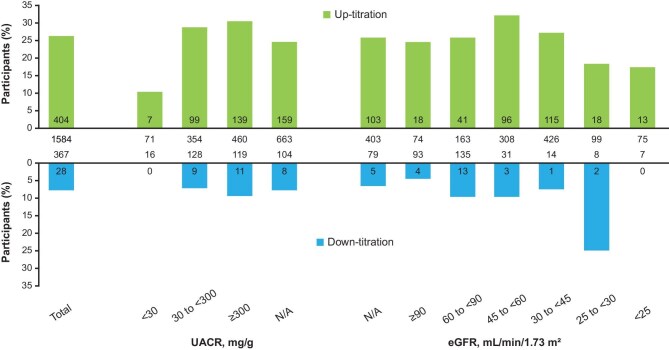
Up-titration and down-titration of finerenone overall and by participant characteristics. Numbers within bars indicate numbers of participants with up- or down-titration. Numbers below or above bars indicate numbers of participants at risk. N/A: not available.

There was no clear relationship between participant characteristics and the use of up- or down-titration (Fig. 6) or its timing (Supplementary Table S2). Sex, CV events and the use of RASis, GLP-1RAs or SGLT2is did not have a major effect on titration (data not shown).

Finerenone was continuously administered (every day for the entire observation period without interruption or premature discontinuation) in 1618 (84.4%) participants for >1 year and was interrupted (not classed as down-titration) in 118 (6.2%) participants, of whom 91 (77.1%) restarted without the simultaneous addition of any other new co-medication, and 27 (22.9%) restarted in combination with other medications. The most common of these were diuretics [12 (0.6%) participants], insulin [10 (0.5%)] and potassium binders [9 (0.5%)] (some participants added more than one new co-medication). In total, 251 (13.1%) participants discontinued finerenone, including temporary discontinuation and those who had not resumed treatment at data cut-off. An AE was listed as a reason for discontinuation in 76 (4.0%) participants. Permanent discontinuation because of an AE was reported for 51 participants, most commonly because of hyperkalaemia [20 (1.0%) participants], participant decision [45 (2.3%)] and physician decision [16 (0.8%)]. The median duration of treatment if participants discontinued was 105 days (IQR 42–203).

### Concomitant medication

**Figure 7:**
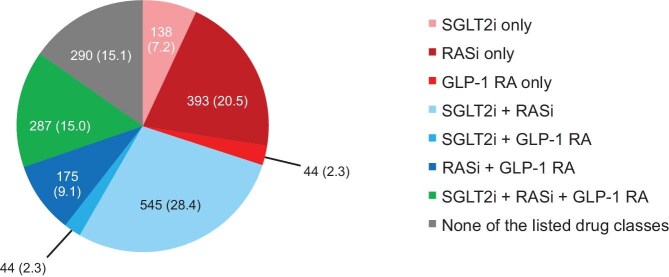
Co-medication with RASi, SGLT2i or GLP-1RA at baseline. Data presented as *n* (%). The combination of SGLT2i, GLP-1RA and non-steroidal MRA has been shown to reduce major adverse CV events in patients with T2D and albuminuria compared with conventional therapy [53]. These classes plus RASi are considered the four pillars of CKD treatment.

In total, 138 (7.2%) participants received SGLT2is only, 393 (20.5%) received RASis only, 44 (2.3%) received GLP-1RAs only, 545 (28.4%) received SGLT2is and RASis, 44 (2.3%) received SGLT2is and GLP-1RAs, 175 (9.1%) received RASis and GLP-1RAs, 287 (15.0%) received all three classes (Fig. 7) and 290 (15.1%) participants received none of these classes ahead of starting finerenone therapy.

In total, 1785 (93.2%) participants had at least one prior and concomitant medication, including RASis in 1400 (73.1%) participants, SGLT2is in 1014 (52.9%) and GLP-1RAs in 550 (28.7%). After the start but before the end of finerenone therapy, 96 (5.0%), 121 (6.3%) and 113 (5.9%) participants began treatment with RASis, SGLT2is and GLP-1RAs, respectively. Concomitant therapies comprised medicines for T2D including insulin and analogues, blood pressure control and lipid modification in 1689 (88.2%), 1628 (85.0%) and 1468 (76.6%) participants, respectively.

At the 12-month follow-up, data were available for 415 participants. RASis, SGLT2is and GLP-1RAs were used by 309 (74.5%), 197 (47.5%) and 154 (37.1%) participants, respectively; 375 (90.4%), 372 (89.6%) and 338 (81.4%) participants received T2D treatment including insulin and analogues, blood pressure control and lipid modification, respectively.

### Safety

TEAEs were reported in 693 (36.2%) participants, serious TEAEs in 190 (9.9%) and TEAEs resulting in death in 10 (0.5%). The most common TEAEs were hyperkalaemia (including those with a blood potassium increase) in 156 (8.1%) participants, urinary tract infection in 76 (4.0%) and urogenital tract haemorrhage in 51 (2.7%) (Table 4). The most common serious TEAEs were hyperkalaemia in 16 (0.8%) participants and renal failure in 15 (0.8%) (Table 3). The incidence of treatment-emergent hyperkalaemia and potassium increase is shown in Table 5.

**Table 4:** Most common TEAEs and serious TEAEs (FAS, *n* = 1916).

Event, *n* (%)	Values
TEAEs (>1.0%)	
Hyperkalaemia^a^	156 (8.1)
Urinary tract infection	76 (4.0)
Urogenital tract haemorrhage^b^	51 (2.7)
Renal failure	23 (1.2)
COVID-19	23 (1.2)
Oedema	22 (1.2)
Hyperuricaemia^c^	22 (1.1)
Dizziness	20 (1.0)
Serious TEAEs (>0.5%)	
Hyperkalaemia^a^	16 (0.8)
Renal failure	15 (0.8)

a Including hyperkalaemia and blood potassium increased.

b Including blood urine present, haematuria, blood urine, red blood cells urine positive and haemorrhagic cystitis.

c Including hyperuricaemia and increased blood uric acid.

**Table 5:** Incidence of hyperkalaemia and potassium levels.

Event, *n* (%)	Participants (*n* = 1916)
TEAE	
Any	148 (7.7)
Serious	15 (0.8)
ADR	
Any	132 (6.9)
Serious	14 (0.7)
Treatment-emergent potassium level	
>5.5 mmol/l	120 (6.3)
>6 mmol/l	28 (1.5)

ADR: adverse drug reaction.

Hyperkalaemia was also assessed as a secondary outcome of the study. In this analysis, of the 156 (8.1%) hyperkalaemia events reported, 20 led to study drug discontinuation, 16 were serious, 5 led to hospitalization and none were fatal.

## DISCUSSION

FINE-REAL is the first global, prospective, observational study investigating the use of a non-steroidal MRA in routine clinical care in participants with CKD and T2D. This interim analysis presents baseline characteristics of the patients enrolled in FINE-REAL and also provides timely real-world data on the dosing, concomitant medications and safety of finerenone in participants with CKD and T2D.

Compared with FIDELITY [26], more participants in FINE-REAL were at moderate KDIGO risk at baseline and fewer were at high risk. This suggests that physicians are prescribing finerenone at an earlier CKD stage in routine practice compared with randomized clinical trials. UACR screening is crucial for early detection and management of albuminuria [44] and is recommended in the American Diabetes Association and KDIGO guidelines [45, 46]. In FINE-REAL, 75% of participants had UACR measurements versus 47% in the Awareness, Detection and Drug Therapy in Type 2 Diabetes and Chronic Kidney Disease (ADD-CKD) study [47] and 9% in the Center for Kidney Disease Research, Education and Hope (HOPE) registry [48]. These results may partly reflect differences in study design and setting: FINE-REAL is an observational trial with primary data generation, HOPE was an electronic medical records–based study (secondary data generation) depending on prior evaluation and recording of data and ADD-CKD was a primary care study (primary data generation).

Most participants (80.8%) started finerenone at 10 mg, and 19.2% started at 20 mg. Consistent with label recommendations [30, 31], 94.5% of participants with baseline eGFR ≥25–<60 ml/min/1.73 m^2^ received the recommended starting dose of 10 mg. However, 54.6% of those with baseline eGFR ≥60 ml/min/1.73 m^2^ also received this dose (10 mg) rather than the recommended 20 mg. The labelling, based on previous study results, recommends increasing the dose to 20 mg if the eGFR decrease is ≤30% and serum potassium is ≤4.8 mmol/l [30, 31], but only 18.4% of participants starting at 10 mg who met these criteria underwent up-titration. Baseline UACR had relatively little effect on the initial dose, and there was a tendency for the 20 mg dose to be prescribed less frequently as baseline serum potassium rose. At 12 months, 64.8% of participants were still receiving 10 mg and only 35.4% were receiving 20 mg. These results suggest a reluctance on the part of many physicians to increase the dose of finerenone as recommended.

Finerenone was well tolerated and safety was consistent with the known safety profile of the drug [30, 31], with serious TEAEs reported in 9.9% of participants. Importantly, the definition of TEAEs included all AEs regardless of any potential relationship to study treatment. In addition, the occurrence of hyperkalaemia was low (8.1%); ≤1 event led to study drug discontinuation, hospitalization or were defined as serious. In participants initiating with 10 mg, the co-occurrence of an eGFR reduction >30% and potassium >4.8 mmol/l was rare (1.2% of participants).

Finerenone was continuously administered in 84.5% of participants, while in 6.2% finerenone treatment was interrupted for any reason—e.g. the occurrence of an AE—and finerenone was permanently discontinued in 13.1% of participants. These data suggest good adherence to finerenone treatment in the study cohort. This is in contrast to recent real-world studies that reported suboptimal adherence to international guidelines for co-medication with RASis, SGLT2is or GLP-1RAs in participants with T2D and CKD [49–51]. In FINE-REAL, RASis, SGLT2is and GLP-1RAs were used by 73.1%, 52.9% and 28.7% of participants, respectively, at baseline and by 74.5%, 47.5% and 37.1% of participants, respectively, at 12 months. It is important to note, however, that not all participants in this interim analysis had completed the 12-month observation period by the cut-off date, and a lower rate of SGLT2i use at 12 months compared with baseline does not necessarily mean that the drug was discontinued. The concomitant use of SGLT2is is of interest because combination therapy with finerenone and an SGLT2i introduced simultaneously was demonstrated in the CONFIDENCE trial to produce a greater reduction in UACR than either treatment alone [52]. Overall, these results suggest that finerenone is and can be successfully combined with other guideline-directed therapies in routine practice.

One of the strengths of FINE-REAL is the diverse participant population, which enhances the generalizability of our findings. FINE-REAL had a broad spectrum of participants, including those with various stages of CKD and multiple comorbidities, such as diabetic retinopathy, diabetic neuropathy, atrial fibrillation and coronary artery disease. This diversity provides a comprehensive understanding of finerenone use in real-world settings. However, FINE-REAL does have some limitations. As an observational study, it is subject to potential biases and confounding factors inherent to real-world research, while the lack of a randomized control group limits the ability to draw definitive conclusions. In addition, the follow-up period, while sufficient to observe outcomes, may not capture long-term effects and rare AEs. Despite these limitations, FINE-REAL adds valuable real-world evidence to the literature to guide and support the use of finerenone in participants with T2D and CKD.

## CONCLUSIONS

FINE-REAL is the first global, prospective, observational study investigating the use of finerenone in routine clinical care, with interim data on nearly 2000 participants and a median follow-up of 9 months. In this diverse and well-represented population, most participants initiated finerenone at 10 mg and remained on this dose; titration to 20 mg, as recommended on the label for most patients, was uncommon. Finerenone was well tolerated and safety was consistent with the known safety profile of the drug [30, 31]. FINE-REAL is ongoing and further results will be published when more data become available. This interim analysis and future data from this ongoing study will help to improve informed decision-making regarding initiation of finerenone in participants with CKD and T2D and improve adherence to the dosing recommendations set out on the product label for finerenone.

## Supplementary Material

sfaf305_Supplemental_File

## Data Availability

The availability of the data underlying this publication will be determined according to Bayer’s commitment to the European Federation of Pharmaceutical Industries and Associations and Pharmaceutical Research and Manufacturers of America principles for responsible clinical trial data sharing, pertaining to scope, time point and process of data access. Bayer commits to sharing upon request from qualified scientific and medical researchers, patient-level clinical trial data, study-level clinical trial data and protocols from clinical trials in patients for medicines and indications approved in the USA and European Union (EU) as necessary for doing legitimate research. This commitment applies to data on new medicines and indications that have been approved by the EU and US regulatory agencies on or after 1 January 2014. Interested researchers can use www.clinicalstudydatarequest.com to request access to anonymized patient-level data and supporting documents from clinical studies to do further research that can help advance medical science or improve patient care. Information on the Bayer criteria for listing studies and other relevant information is provided in the study sponsors section of the portal.

## References

[1] Li H, Wanhong L, Ani W et al. Changing epidemiology of chronic kidney disease as a result of type 2 diabetes mellitus from 1990 to 2017: estimates from global burden of disease 2017. J Diabetes Investig 2021;12:346–56. 10.1111/jdi.13355 PMC7926234 32654341

[2] Kumar M, Dev S, Khalid MU et al. The bidirectional link between diabetes and kidney disease: mechanisms and management. Cureus 2023;15:e45615. 10.7759/cureus.45615 37868469 PMC10588295

[3] Chung H, Crowe CL, Kong SX et al. Descriptive study of the economic burden among patients with type 2 diabetes mellitus, chronic kidney disease, and chronic kidney disease and type 2 diabetes mellitus in a large US commercially insured population. J Manag Care Spec Pharm 2023;29:80–9. 10.18553/jmcp.2023.29.1.80 36580126 PMC10387965

[4] Wan EYF, Chin WY, Yu EYT et al. The impact of cardiovascular disease and chronic kidney disease on life expectancy and direct medical cost in a 10-year diabetes cohort study. Diabetes Care 2020;43:1750–8. 10.2337/dc19-2137 32457057

[5] Bello AK, Okpechi IG, Levin A et al. An update on the global disparities in kidney disease burden and care across world countries and regions. Lancet Glob Health 2024;12:e382–95. 10.1016/S2214-109X(23)00570-3 38365413

[6] World Health Organization . Diabetes. 2024. https://www.who.int/news-room/fact-sheets/detail/diabetes (18 November 2024, date last accessed).

[7] Nelson RG, Grams ME, Ballew SH et al. Development of risk prediction equations for incident chronic kidney disease. JAMA 2019;322:2104–14. 10.1001/jama.2019.17379 31703124 PMC6865298

[8] Bakris GL, Agarwal R, Anker SD et al. Effect of finerenone on chronic kidney disease outcomes in type 2 diabetes. N Engl J Med 2020;383:2219–29. 10.1056/NEJMoa2025845 33264825

[9] Chou CL, Chiu HW, Hsu YH et al. Impact of chronic kidney disease and end-stage renal disease on the mid-term adverse outcomes in diabetic patients with cardiovascular diseases. Sci Rep 2024;14:15770. 10.1038/s41598-024-66655-0 38982230 PMC11233494

[10] Xing J, Huang L, Ren W et al. Risk factors for rapid kidney function decline in diabetes patients. Ren Fail 2024;46:2398188. 10.1080/0886022X.2024.2398188 39258389 PMC11391878

[11] Branch M, German C, Bertoni A et al. Incremental risk of cardiovascular disease and/or chronic kidney disease for future ASCVD and mortality in patients with type 2 diabetes mellitus: ACCORD trial. J Diabetes Complications 2019;33:468–72. 10.1016/j.jdiacomp.2019.04.004 31088728

[12] Swamy S, Noor SM, Mathew RO. Cardiovascular disease in diabetes and chronic kidney disease. J Clin Med 2023;12:6984. 10.3390/jcm12226984 38002599 PMC10672715

[13] Rai NK, Wang Z, Drawz PE et al. CKD progression risk and subsequent cause of death: a population-based cohort study. Kidney Med 2023;5:100604. 10.1016/j.xkme.2023.100604 36970224 PMC10034498

[14] Zoccali C, Mallamaci F, Adamczak M et al. Cardiovascular complications in chronic kidney disease: a review from the European renal and cardiovascular medicine working group of the European Renal Association. Cardiovasc Res 2023;119:2017–32. 10.1093/cvr/cvad083 37249051 PMC10478756

[15] Burnier M, Damianaki A. Hypertension as cardiovascular risk factor in chronic kidney disease. Circ Res 2023;132:1050–63. 10.1161/CIRCRESAHA.122.321762 37053276

[16] Sattar N, Presslie C, Rutter MK et al. Cardiovascular and kidney risks in individuals with type 2 diabetes: contemporary understanding with greater emphasis on excess adiposity. Diabetes Care 2024;47:531–43. 10.2337/dci23-0041 38412040

[17] Baaten CCFMJ, Vondenhoff S, Noels H. Endothelial cell dysfunction and increased cardiovascular risk in patients with chronic kidney disease. Circ Res 2023;132:970–92. 10.1161/CIRCRESAHA.123.321752 37053275 PMC10097498

[18] Wang N, Zhang C. Oxidative stress: a culprit in the progression of diabetic kidney disease. Antioxidants 2024;13:455. 10.3390/antiox13040455 38671903 PMC11047699

[19] Fox CS, Matsushita K, Woodward M et al. Associations of kidney disease measures with mortality and end-stage renal disease in individuals with and without diabetes: a meta-analysis. Lancet 2012;380:1662–73. 10.1016/S0140-6736(12)61350-6 23013602 PMC3771350

[20] van der Velde M, Matsushita K, Coresh J et al. Lower estimated glomerular filtration rate and higher albuminuria are associated with all-cause and cardiovascular mortality. A collaborative meta-analysis of high-risk population cohorts. Kidney Int 2011;79:1341–52. 10.1038/ki.2010.536 21307840

[21] Lv JC, Zhang LX. Prevalence and disease burden of chronic kidney disease. Adv Exp Med Biol 2019;1165:3–15. 10.1007/978-981-13-8871-2_1 31399958

[22] Aroor AR, Habibi J, Nistala R et al. Diet-induced obesity promotes kidney endothelial stiffening and fibrosis dependent on the endothelial mineralocorticoid receptor. Hypertension 2019;73:849–58. 10.1161/HYPERTENSIONAHA.118.12198 30827147 PMC6448566

[23] Barrera-Chimal J, Jaisser F. Vascular mineralocorticoid receptor activation and disease. Exp Eye Res 2019;188:107796. 10.1016/j.exer.2019.107796 31521629

[24] Filippatos G, Anker SD, Böhm M et al. A randomized controlled study of finerenone vs. eplerenone in patients with worsening chronic heart failure and diabetes mellitus and/or chronic kidney disease. Eur Heart J 2016;37:2105–14. 10.1093/eurheartj/ehw132 27130705 PMC4946749

[25] Bansal S, Canziani MEF, Birne R et al. Finerenone cardiovascular and kidney outcomes by age and sex: FIDELITY post hoc analysis of two phase 3, multicentre, double-blind trials. BMJ Open 2024;14:e076444. 10.1136/bmjopen-2023-076444 PMC10952937 38508632

[26] Agarwal R, Filippatos G, Pitt B et al. Cardiovascular and kidney outcomes with finerenone in patients with type 2 diabetes and chronic kidney disease: the FIDELITY pooled analysis. Eur Heart J 2022;43:474–84. 10.1093/eurheartj/ehab777 35023547 PMC8830527

[27] Kolkhof P, Delbeck M, Kretschmer A et al. Finerenone, a novel selective nonsteroidal mineralocorticoid receptor antagonist protects from rat cardiorenal injury. J Cardiovasc Pharmacol 2014;64:69–78. 10.1097/FJC.0000000000000091 24621652

[28] Pitt B, Kober L, Ponikowski P et al. Safety and tolerability of the novel non-steroidal mineralocorticoid receptor antagonist BAY 94-8862 in patients with chronic heart failure and mild or moderate chronic kidney disease: a randomized, double-blind trial. Eur Heart J 2013;34:2453–63. 10.1093/eurheartj/eht187 23713082 PMC3743070

[29] Pitt B, Filippatos M, Agarwal R et al. Cardiovascular events with finerenone in kidney disease and type 2 diabetes. N Engl J Med 2021;385:2252–63. 10.1056/NEJMoa2110956 34449181

[30] Bayer HealthCare Pharmaceuticals . KERENDIA (finerenone) tablets, for oral use: US prescribing information. 2021. https://labeling.bayerhealthcare.com/html/products/pi/Kerendia_PI.pdf (23 July 2024, date last accessed).

[31] Bayer HealthCare Pharmaceuticals . Kerendia summary of product characteristics. 2022. https://www.ema.europa.eu/en/documents/product-information/kerendia-epar-product-information_en.pdf (27 July 2024, date last accessed).

[32] de Boer IH, Khunti K, Sadusky T et al. Diabetes management in chronic kidney disease: a consensus report by the American Diabetes Association (ADA) and Kidney Disease: Improving Global Outcomes (KDIGO). Diabetes Care 2022;45:3075–90. 10.2337/dci22-0027 36189689 PMC9870667

[33] Blonde L, Umpierrez GE, Reddy SS et al. American Association of Clinical Endocrinology clinical practice guideline: developing a diabetes mellitus comprehensive care plan—2022 update. Endocr Pract 2022;28:923–1049. 10.1016/j.eprac.2022.08.002 35963508 PMC10200071

[34] Desai NR, Navaneethan SD, Nicholas SB et al. Design and rationale of FINE-REAL: a prospective study of finerenone in clinical practice. J Diabetes Complications 2023;37:108411. 10.1016/j.jdiacomp.2023.108411 36857997

[35] Nicholas SB, Correa-Rotter R, Desai NR et al. First interim results from FINE-REAL: a prospective, non-interventional, phase 4 study providing insights into the use and safety of finerenone in a routine clinical setting. J Nephrol 2024;37:2223–32. 10.1007/s40620-024-02070-y 39340711 PMC11649709

[36] Quaggin SE, Palevsky PM. Removing race from kidney disease diagnosis. J Am Soc Nephrol 2021;32:2987–9. 10.1681/ASN.2021091284 34753827 PMC8638407

[37] Inker LA, Eneanya ND, Coresh J et al. New creatinine- and cystatin C-based equations to estimate GFR without race. N Engl J Med 2021;385:1737–49. 10.1056/NEJMoa2102953 34554658 PMC8822996

[38] Levey AS, Stevens LA, Schmid CH et al. A new equation to estimate glomerular filtration rate. Ann Intern Med 2009;150:604–12. 10.7326/0003-4819-150-9-200905050-00006 19414839 PMC2763564

[39] Levey AS, Eckardt KU, Dorman NM et al. Nomenclature for kidney function and disease: report of a Kidney Disease: Improving Global Outcomes (KDIGO) consensus conference. Kidney Int 2020;97:1117–29. 10.1016/j.kint.2020.02.010 32409237

[40] Brown EG, Wood L, Wood S. The Medical Dictionary for Regulatory Activities (MedDRA). Drug Saf 1999;20:109–17. 10.2165/00002018-199920020-00002 10082069

[41] Filippatos G, Anker SD, Agarwal R et al. Finerenone and cardiovascular outcomes in patients with chronic kidney disease and type 2 diabetes. Circulation 2021;143:540–52. 10.1161/CIRCULATIONAHA.120.051898 33198491 PMC7864612

[42] US Department of Health and Human Services . Code of Federal Regulations. 21 CFR § 11.1. 2012. https://www.ecfr.gov/current/title-21/chapter-I/subchapter-A/part-11 (24 July 2024, date last accessed).

[43] Aalen OO, Johansen S. An empirical transition matrix for non-homogeneous Markov chains based on censored observations. Scand J Stat 1978;5:141–50. http://www.jstor.org/stable/4615704

[44] Christofides EA, Desai N. Optimal early diagnosis and monitoring of diabetic kidney disease in type 2 diabetes mellitus: addressing the barriers to albuminuria testing. J Prim Care Community Health 2021;12:21501327211003683. 10.1177/21501327211003683 33749371 PMC7983418

[45] Kidney Disease: Improving Global Outcomes CKD Work Group . KDIGO 2024 clinical practice guideline for the evaluation and management of chronic kidney disease. Kidney Int 2024;105(4 Suppl):S117–314. 10.1016/j.kint.2023.10.018 38490803

[46] American Diabetes Association Professional Practice Committee . 11. Chronic kidney disease and risk management: standards of care in diabetes—2025. Diabetes Care 2025;48(Suppl 1):S239–51. 10.2337/dc25-S011 39651975 PMC11635029

[47] Szczech LA, Stewart RC, Su HL et al. Primary care detection of chronic kidney disease in adults with type-2 diabetes: the ADD-CKD Study (awareness, detection and drug therapy in type 2 diabetes and chronic kidney disease). PLoS One 2014;9:e110535. 10.1371/journal.pone.0110535 25427285 PMC4245114

[48] Tuttle KR, Alicic RZ, Kenrik Duru O et al. Clinical characteristics of and risk factors for chronic kidney disease among adults and children: an analysis of the CURE-CKD registry. JAMA Netw Open 2019;2:e1918169. 10.1001/jamanetworkopen.2019.18169 31860111 PMC6991307

[49] Nicholas SB, Daratha KB, Alicic RZ et al. Prescription of guideline-directed medical therapies in patients with diabetes and chronic kidney disease from the CURE-CKD Registry, 2019–2020. Diabetes Obes Metab 2023;25:2970–9. 10.1111/dom.15194 37395334

[50] Lim CE, Pasternak B, Eliasson et al. Use of sodium–glucose co-transporter 2 inhibitors and glucagon-like peptide-1 receptor agonists according to the 2019 ESC guidelines and the 2019 ADA/EASD consensus report in a national population of patients with type 2 diabetes. Eur J Prev Cardiol 2023;30:634–43. 10.1093/eurjpc/zwac315 36582120

[51] Jeong SJ, Lee SE, Shin DH et al. Barriers to initiating SGLT2 inhibitors in diabetic kidney disease: a real-world study. BMC Nephrol 2021;22:177. 10.1186/s12882-021-02381-3 33990175 PMC8122538

[52] Agarwal R, Green JB, Heerspink HJL et al. Finerenone with empagliflozin in chronic kidney disease and type 2 diabetes. N Engl J Med 2025;393:533–43. 10.1056/NEJMoa2410659 40470996

[53] Neuen BL, Heerspink HJL, Vart P et al. Estimated lifetime cardiovascular, kidney, and mortality benefits of combination treatment with SGLT2 inhibitors, GLP-1 receptor agonists, and nonsteroidal MRA compared with conventional care in patients with type 2 diabetes and albuminuria. Circulation 2024;149:450–62. 10.1161/CIRCULATIONAHA.123.067584 37952217

