# Supplementary material for: Dosing, treatment patterns and safety of finerenone use in routine care: an interim analysis of the prospective, real-world and observational FINE-REAL study
Source: Clin Kidney J. 2025 Oct 6;18(11):sfaf305. doi: 10.1093/ckj/sfaf305 (PMC12598660; doi:10.1093/ckj/sfaf305)
Supplement: sfaf305_Supplemental_File [file sfaf305_supplemental_file.docx]

**SUPPLEMENTARY MATERIALS**

**
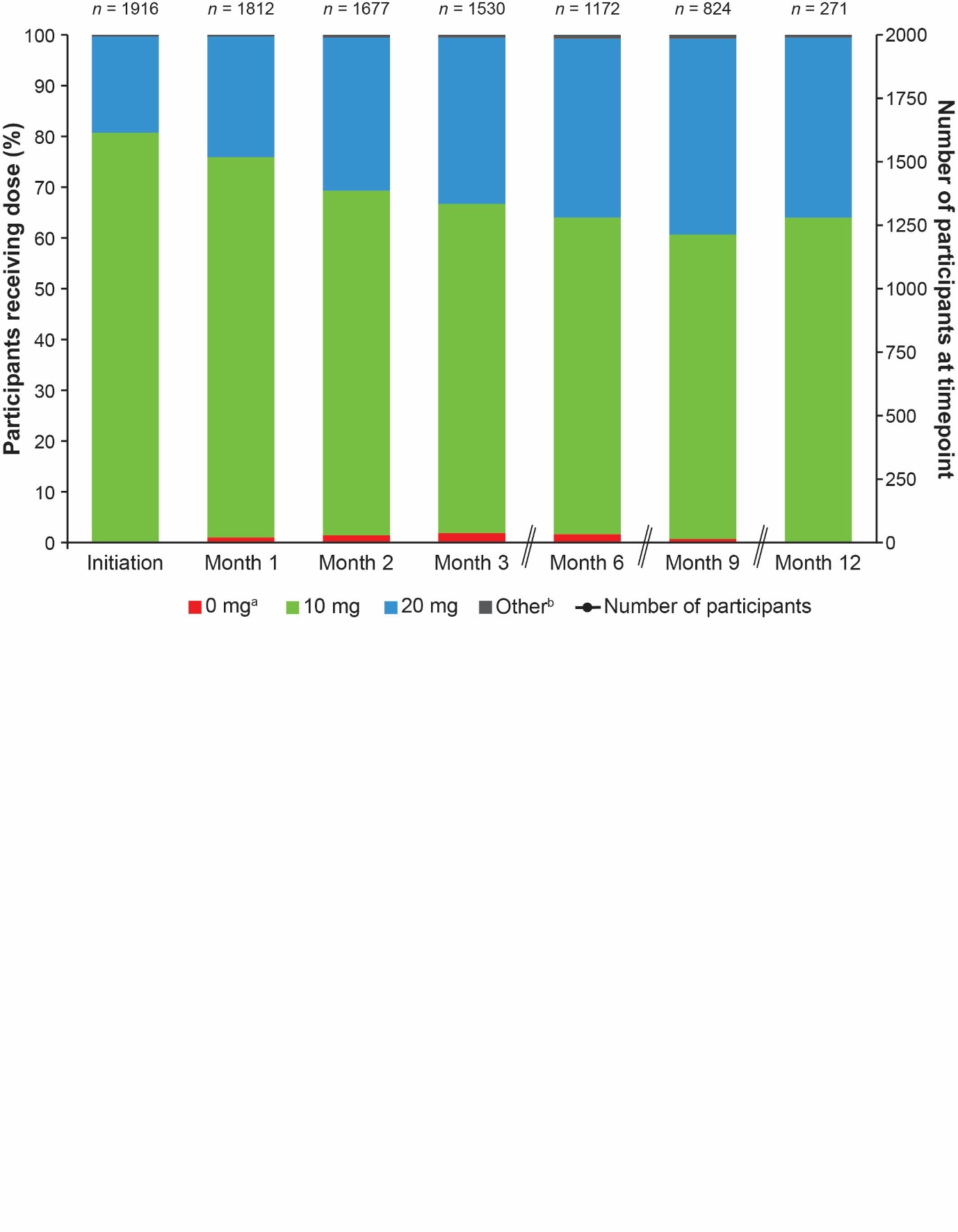
Supplementary Figure S1:** Dosing of finerenone in the overall population during the study. ^a^Participants in whom finerenone was interrupted and restarted later. ^b^Some physicians composed their own treatment regimens, such as 10 mg of finerenone four times per week.

**Supplementary Table S1: Availability of eGFR and UACR values at baseline.**

| **Number of participants** | **FAS (*n* = 1916)** |
| --- | --- |
| No eGFR value | 82 (4.3) |
| Baseline eGFR after start of finerenone | 84 (4.4) |
| Baseline eGFR before or at start of finerenone | 1750 (91.3) |
| Baseline eGFR >8 weeks before start of finerenone | 190 (9.9) |
| Baseline eGFR >6 months before start of finerenone | 38 (2.0) |
|  |  |
| No UACR value | 411 (21.5) |
| Baseline UACR after start of finerenone | 63 (3.3) |
| Baseline UACR before or at start of finerenone | 1442 (75.3) |
| Baseline UACR >8 weeks before start of finerenone | 143 (7.5) |
| Baseline UACR >6 months before start of finerenone | 43 (2.2) |
| Baseline UACR and eGFR values at start of finerenone | 1410 (73.6) |

eGFR, estimated glomerular filtration rate; FAS, full analysis set; UACR, urine albumin:creatinine ratio.

**Supplementary Table S2: Median time (days) to up- or down-titration of finerenone across participant subgroups (FAS).**

| **Characteristic** | **Median time from first dose of finerenone 10 mg to up-titration to 20 mg (IQR)** | **Median time from first dose of finerenone 20 mg to down-titration to 10 mg (IQR)** |
| --- | --- | --- |
| All participants | *n* = 404  47.0 (30.0 to 108.5) | *n* = 28  58.0 (30.0 to 139.0) |
| eGFR at initiation, mL/min/1.73 m² | | |
| No eGFR available | *n* = 103  77.0 (39.0 to 164.0) | *n* = 5  68.0 (48.0 to 217.0) |
| ≥90 | *n* = 18  37.0 (30.0 to 60.0) | *n* = 4  128.5 (68.0 to 139.0) |
| 60 to <90 | *n* = 41  53.0 (30.0 to 99.0) | *n* = 13  43.0 (29.0 to 114.0) |
| 45 to <60 | *n* = 96  39.0 (29.0 to 114.0) | *n* = 3  31.0 (28.0 to 127.0) |
| 30 to <45 | *n* = 115  43.0 (30.0 to 99.0) | *n* = 1  39.0 (39.0 to 39.0) |
| 25 to <30 | *n* = 18  34.5 (30.0 to 44.0) | *n* = 2  152.5 (25.0 to 280.0) |
| <25 | *n* = 13  36.0 (29.0 to 62.0) | 0 |
| UACR at initiation, mg/g |  |  |
| No UACR available | *n* = 159  64.0 (34.0 to 135.0) | *n* = 8  58.0 (42.5 to 192.0) |
| ≥300 | *n* = 139  37.0 (29.0 to 103.0) | *n* = 11  104.0 (35.0 to 253.0) |
| 30 to <300 | *n* = 99  43.0 (29.0 to 95.0) | *n* = 9  31.0 (28.0 to 114.0) |
| <30 | *n* = 7  83.0 (35.0 to 283.0) | 0 |

eGFR, estimated glomerular filtration rate; FAS, full analysis set; IQR, interquartile range; UACR, urine albumin:creatinine ratio.
